# Shear Wave Elastography in Musculoskeletal Imaging: A Narrative Review

**DOI:** 10.3390/jcm15124843

**Published:** 2026-06-22

**Authors:** Enes Gurun, Mesut Ozturk, Mustafa Basaran, Ahmet Emin Okutan

**Affiliations:** 1 Department of Radiology, Faculty of Medicine, Samsun University, 55080 Samsun, Türkiye; 2 Department of Orthopedics and Traumatology, Faculty of Medicine, Samsun University, 55080 Samsun, Türkiye

**Keywords:** sonoelastography, shear wave elastography, elasticity imaging techniques, musculoskeletal system, ultrasonography, tendons, skeletal muscle, peripheral nerves

## Abstract

Shear wave elastography (SWE) is an increasingly investigated ultrasound-based technique in musculoskeletal imaging that provides quantitative information on tissue stiffness and biomechanical properties. This narrative review aims to summarize the basic principles, technical considerations, current clinical applications, limitations, and future perspectives of SWE in musculoskeletal imaging. Unlike conventional grayscale and Doppler ultrasonography, which mainly assess morphology and vascularity, SWE may provide additional functional information in major musculoskeletal tissues, including tendons and ligaments, skeletal muscles, peripheral nerves, fibrocartilaginous structures, plantar fascia, and selected soft tissue lesions. Current evidence suggests potential roles for SWE in detecting early biomechanical alterations, assessing disease severity, differentiating symptomatic from asymptomatic tissues, and monitoring response to treatment or rehabilitation. However, musculoskeletal tissues are anisotropic, viscoelastic, and position-dependent; as a result, SWE measurements are influenced by acquisition-related factors, tissue biomechanics, positioning and loading conditions, region of interest (ROI) placement, tissue depth, and device-related variability. For this reason, SWE findings should not be interpreted as standalone diagnostic criteria but should be considered together with clinical findings, conventional ultrasonography, MRI, electrophysiology, histopathology, and patient-centered outcomes when appropriate. This review highlights the need for tissue-specific measurement protocols, standardized reporting, normative reference data, inter-vendor harmonization, and longitudinal validation against clinically meaningful outcomes before SWE can be more reliably integrated into routine musculoskeletal imaging and rehabilitation practice.

## 1. Introduction

Musculoskeletal ultrasonography (US) has become an important part of current clinical practice because it is widely available, is inexpensive, provides high spatial resolution, allows real-time dynamic assessment, and does not involve ionizing radiation. In particular, the routine use of high-frequency probes has made it easier to evaluate superficial structures such as tendons, ligaments, muscles, peripheral nerves, and fascia in detail. However, conventional grayscale and Doppler US are mainly based on morphological and vascular features and may be limited in directly demonstrating the mechanical properties of tissues. Therefore, particularly in early-stage or subclinical musculoskeletal disorders, and in cases where structural imaging findings do not fully correlate with symptoms, subtle changes in tissue mechanics may not be detected by conventional imaging [1,2,3,4].

Elastography refers to a group of ultrasound-based techniques designed to evaluate the mechanical properties of tissues. In musculoskeletal imaging, the most commonly discussed approaches include strain or compression elastography, acoustic radiation force impulse (ARFI) imaging, point shear wave elastography (pSWE), and two-dimensional shear wave elastography (2D-SWE). Strain elastography mainly assesses relative tissue deformation induced by manual compression or physiological motion and is therefore more operator-dependent and semiquantitative. ARFI-based techniques use focused acoustic radiation force to generate localized tissue displacement. Point SWE provides a quantitative shear wave velocity measurement from a predefined sampling region, whereas 2D-SWE generates a color-coded elastographic map over a larger field of view and allows quantitative measurements within selected regions of interest. These techniques differ in excitation method, output parameters, reproducibility, artifacts, and clinical interpretation, which is particularly important in musculoskeletal tissues because of anisotropy, viscoelasticity, and position-dependent tissue tension [3,4,5,6]. The main differences among elastography techniques used in musculoskeletal imaging are summarized in Table 1.

SWE has added a complementary dimension to conventional US by allowing quantitative assessment of tissue stiffness. By measuring the propagation of shear waves generated within tissue by acoustic radiation force, SWE provides objective biomechanical information through shear wave velocity or stiffness-related parameters. Compared with strain elastography, which is a semiquantitative method, SWE is quantitative, less operator-dependent, and more reproducible, making it particularly attractive for musculoskeletal applications. In this way, the role of US has expanded beyond structural assessment toward the evaluation of the functional and mechanical properties of soft tissues [3,4,5].

In recent years, SWE has been increasingly investigated for the evaluation of many musculoskeletal structures, including tendons, the rotator cuff, skeletal muscles, peripheral nerves, plantar fascia, cartilage, and some soft tissue masses. Recent reviews suggest that SWE may be useful not only for diagnosis but also for disease monitoring in areas such as tendinopathies, muscle spasticity, assessment of nerve stiffness, tissues related to knee osteoarthritis, plantar fasciitis, and the differential diagnosis of soft tissue tumors. However, this rapidly growing literature shows considerable methodological heterogeneity. Differences in measurement protocols, extremity and joint position, probe placement and compression, region of interest selection, tissue anisotropy, reported parameters, and inter-vendor variability make direct comparison across studies difficult and limit the interpretation of reported cutoff values and clinical performance data [7,8,9,10,11,12,13,14,15].

**Figure 1:**
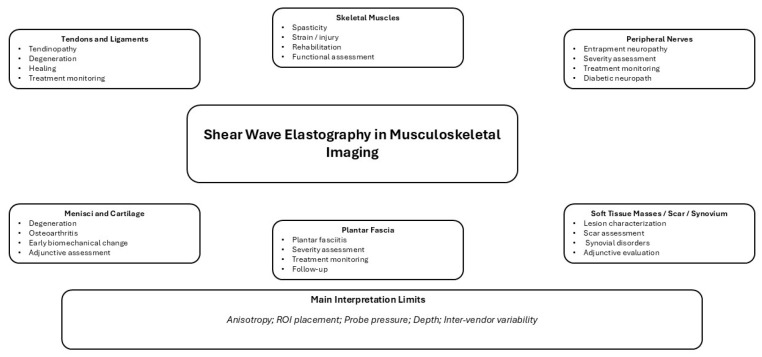
Schematic overview of musculoskeletal applications and interpretation limits of shear wave elastography (SWE). SWE has been investigated in tendons and ligaments, skeletal muscles, peripheral nerves, menisci, cartilage, plantar fascia, and selected soft tissue lesions. Its potential clinical roles include adjunctive diagnosis, severity assessment, treatment monitoring, rehabilitation follow-up, and biomechanical biomarker development. Interpretation should consider tissue anisotropy, loading condition, probe pressure, ROI placement, measurement depth, and inter-vendor variability.

SWE has been increasingly applied across a broad range of musculoskeletal tissues; however, differences in acquisition protocols, reported parameters, and clinical endpoints limit direct comparison across studies. These inconsistencies limit the comparability of reported SWE values and make it difficult to interpret proposed tissue-specific reference values, cutoff thresholds, and clinical performance data across studies. Therefore, a narrative synthesis is needed to clarify how technical determinants, tissue-specific applications, and current evidence gaps should be considered when interpreting musculoskeletal shear wave elastography (MSK-SWE) findings. This review summarizes the basic principles of SWE, key technical considerations affecting measurement reliability, and current and emerging musculoskeletal applications, with emphasis on clinical utility, limitations, standardization needs, and future directions. A schematic overview of the main musculoskeletal applications of SWE and their key interpretation limits is provided in Figure 1.

## 2. Literature Search Strategy

This article was conceived as a narrative review informed by a targeted literature search. The search was primarily conducted in PubMed/MEDLINE for articles published up to May 2026, with additional screening of the reference lists of selected articles to identify further relevant publications. Search terms included combinations of “shear wave elastography,” “sonoelastography,” “musculoskeletal,” “tendon,” “ligament,” “skeletal muscle,” “peripheral nerve,” “carpal tunnel syndrome,” “meniscus,” “cartilage,” “plantar fascia,” “soft tissue mass,” “technical considerations,” “artifact,” “standardization,” “reproducibility,” and “reliability.” The complete search strategy, including the main search terms and an example search string, is provided in Supplementary Table S1.

The review focused on articles published within the last 10 years because MSK-SWE is a rapidly evolving field, and more recent studies better reflect current SWE technologies, acquisition protocols, device software, reporting practices, and clinical applications. The literature search and initial screening were performed by one author and verified by a second author, while potentially relevant articles were discussed among the authors for final inclusion.

Original research articles were prioritized for tissue-specific applications. Prospective and retrospective clinical studies, diagnostic accuracy studies, interventional follow-up studies, reliability studies, review articles, systematic reviews, scoping reviews, technical papers, and guideline/consensus documents were considered when relevant to the technical, methodological, or clinical aspects of MSK-SWE. Given the narrative design, the evidence was synthesized qualitatively, and no quantitative meta-analysis was performed. The available evidence was organized according to major anatomical and clinical domains, including tendons and ligaments, skeletal muscles, peripheral nerves, menisci, cartilage, plantar fascia, soft tissue masses, and other emerging applications. Study selection was guided by clinical relevance, methodological contribution, tissue-specific representativeness, and the ability of each study to illustrate key technical or clinical aspects of MSK-SWE. Because the search strategy was primarily based on PubMed/MEDLINE and reference-list screening, relevant studies indexed only in other databases may have been missed. This should be considered a methodological limitation of the present narrative review.

## 3. Basic Principles of Shear Wave Elastography

SWE is an ultrasound-based imaging technique that quantitatively assesses the stiffness of biological tissues by analyzing the propagation of shear waves generated within the tissue. Unlike conventional grayscale US, SWE provides information about the biomechanical properties of the tissue of interest. Its basic principle is based on measuring the propagation speed of shear waves within the target tissue. In soft tissues, faster shear wave propagation generally indicates greater stiffness, whereas slower propagation indicates greater tissue compliance. Therefore, in musculoskeletal imaging, SWE allows the evaluation of not only the morphological but also the functional and mechanical properties of structures [3,4,5].

The physical basis of SWE relies on local tissue displacement generated by acoustic radiation force. Focused ultrasound “push” pulses produce a brief mechanical force within the target tissue, resulting in shear waves that propagate perpendicular to the original ultrasound beam. The propagation of these waves is tracked using ultrafast imaging techniques, thereby providing quantitative data on the mechanical properties of the tissue. In clinical practice, SWE outputs may be reported directly as shear wave velocity in meters per second (m/s), while some systems also display stiffness-derived parameters in kilopascals (kPa). However, conversion from m/s to kPa is based on ideal assumptions that the tissue is linear, elastic, homogeneous, and isotropic. Because musculoskeletal tissues often do not fully meet these assumptions owing to anisotropy, viscoelasticity, heterogeneity, and loading-dependent mechanical behavior, kPa values should be interpreted cautiously, and direct reporting of shear wave velocity may be more appropriate in many musculoskeletal applications [3,6,9,16].

Strain elastography evaluates relative tissue deformation after manual compression or physiological motion and therefore provides mainly qualitative or semiquantitative information. In contrast, SWE generates shear waves using acoustic radiation force produced by the system rather than manual compression and may provide more objective, quantitative, and reproducible measurements. For this reason, SWE offers important advantages over strain elastography in musculoskeletal applications, where small mechanical differences may have clinical relevance [4,5,6,17,18].

Nevertheless, the application of SWE to musculoskeletal tissues requires particular attention. Structures such as tendons, ligaments, muscles, and peripheral nerves show marked anisotropy, viscoelasticity, and position-dependent mechanical variability. Therefore, probe orientation, transducer compression, ROI selection, patient position, joint angle, tissue tension, and inter-vendor algorithmic differences may affect measurement results. These variables represent key challenges for reproducibility, comparability, and clinical interpretation in MSK-SWE studies [7,10,11,15].

Therefore, SWE measurements should not be interpreted as absolute quantitative parameters independent of technical and biomechanical variables. The obtained values should be evaluated within the relevant anatomical, biomechanical, technical, and device-specific context. Understanding the basic physical principles and measurement assumptions of SWE is essential for the reliable integration of the method into clinical practice [3,4,19].

## 4. Technical Considerations in Musculoskeletal SWE

One of the main limitations in musculoskeletal SWE studies is methodological heterogeneity. Across studies, not only patient populations and the tissues examined but also measurement protocols, device settings, probe placement, output metrics, and assessment conditions vary considerably. This makes it difficult to directly compare values obtained from different studies and reduces the generalizability of reported cutoff values. Therefore, considering technical variables in the interpretation of musculoskeletal SWE data is not merely a methodological issue but a fundamental requirement for measurement reliability and clinical relevance [1,2,3,4,5]. The main technical factors affecting MSK-SWE measurements are summarized in Table 2.

### 4.1. Anisotropy and Probe Orientation

Musculoskeletal tissues, including tendons, ligaments, skeletal muscles, and peripheral nerves, show marked anisotropic properties. Because the mechanical behavior of fibrous structures and the propagation of shear waves vary according to fiber orientation, the orientation of the probe relative to the tissue directly affects measurement results. Shear waves may travel at different speeds along the long and short axes of fibers; therefore, measurements obtained without standardized probe placement are not directly comparable. This is particularly relevant when comparing longitudinal and transverse SWE acquisitions. Longitudinal imaging, in which the probe is aligned parallel to the dominant fiber or fascicular direction, may better reflect stiffness along the main mechanical axis of tendons, ligaments, muscles, or nerves. In contrast, transverse measurements may be more affected by anisotropy, partial volume effects, adjacent tissues, and differences in shear wave propagation across fibers. Therefore, longitudinal and transverse SWE values should not be used interchangeably. The acquisition plane should be standardized within each study and clearly reported, especially when comparing values across patients, follow-up examinations, or different studies [3,5,7,15,19].

### 4.2. Transducer Pressure

Transducer compression is one of the most important operator-dependent variables affecting SWE measurements. Even minimal additional pressure applied to the tissue with the probe may artificially increase local tissue stiffness and lead to overestimated measurements. This effect is particularly pronounced in thin and superficial structures such as superficial tendons, the plantar fascia, and peripheral nerves. Therefore, during SWE examination, light and consistent probe contact should be maintained as much as possible, and unnecessary compression that may cause tissue deformation should be avoided [11,20,21].

### 4.3. ROI Placement and Measurement Depth

ROI selection and placement also directly affect measurement reliability. The part of the target tissue in which the ROI is placed, its relationship with surrounding tissues, its size, and the number of repeated measurements may all contribute to variability in the results. In heterogeneous tissues or lesion areas, the use of a very small ROI may cause sampling error, whereas the use of a very large ROI may include adjacent tissues in the measurement. In measurements performed near regions such as the myotendinous junction, bone interface, or fascial borders, failure to consider adjacent anatomy may lead to misinterpretation. Therefore, ROI selection should be anatomically justified and standardized as much as possible [3,9,22,23,24].

The depth of the examined tissue is another important technical factor affecting the detectability of shear wave propagation and elastogram quality. In deeper tissues, attenuation of acoustic energy, reduced signal-to-noise ratio, and difficulty in tracking shear waves may decrease measurement reliability. This issue is particularly relevant for deep muscles, large musculotendinous units, and structures located beneath thick subcutaneous adipose tissue. In such settings, attenuation of the push pulse and tracking beam may reduce shear wave amplitude and elastogram stability, resulting in wider measurement variability or unreliable values. Therefore, measurement depth, transducer frequency, focal zone, elastogram quality, and the amount of overlying soft tissue should be considered when interpreting SWE data from deeper musculoskeletal structures [15,20,21].

### 4.4. Joint Position and Muscle Activation

Patient position and joint angle may markedly affect SWE measurements by altering passive tissue tension. Because tendons, ligaments, muscles, and peripheral nerves are exposed to different degrees of tension in different joint positions, measuring the same structure at different angles may produce different stiffness values. This is particularly relevant in anatomical regions where muscle relaxation, passive tension, or nerve/tendon tension can easily change. Therefore, patient position, the angle of the examined joint, and whether the extremity is weight-bearing should be clearly defined; when comparisons are made within the same patient or across studies, measurement conditions should be kept consistent [10,25,26,27,28].

In muscle SWE examinations, it is particularly important to specify whether the measurement is performed at rest, under passive tension, or during active contraction. Muscle stiffness physiologically increases during contraction and may also change markedly under passive tension. Therefore, the functional state in which the measurement was obtained should be clearly reported for muscle SWE data to be interpretable. This is also relevant for tendon and musculotendinous unit assessments because muscle activity may indirectly affect tendon stiffness. If the state of the muscle at the time of measurement is not standardized, wide value ranges may be observed even in the same anatomical region [3,5,9,11,29,30].

### 4.5. Vendor Variability and Reproducibility

Inter-vendor differences are among the main methodological issues limiting the standardization of SWE in the musculoskeletal system. The acoustic radiation force or push pulse characteristics, methods of shear wave generation and tracking, signal-processing algorithms, elastogram reconstruction methods, and reported elastography parameters used by different manufacturers may produce different numerical values for the same tissue. Therefore, shear wave velocity (m/s) or elastic modulus (kPa) values obtained with a particular device should not be directly compared with values obtained using a device from another manufacturer [31,32,33]. Similarly, diagnostic cutoff values derived from a specific SWE platform, probe, preset, or software version should not be generalized to other systems unless they have been externally validated under comparable acquisition conditions. In multicenter studies and meta-analyses in particular, vendor variability is an important source of outcome heterogeneity [7,9,10,15,34]. These technical differences also play an important role in the lack of universal cutoff values for the musculoskeletal system.

The quantitative and relatively more objective nature of SWE does not always imply high reproducibility [4]. Intraobserver, interobserver, and interday reliability may vary depending on the tissue examined, anatomical location, device used, probe placement, operator experience, ROI definition, and measurement protocol [20]. Studies on musculoskeletal tissues, particularly muscles, tendons, and peripheral nerves, have shown that although SWE measurements generally provide promising reliability values, the results are not fully homogeneous [10,35,36,37,38]. Limited sample sizes in reliability studies, differences in devices and protocols, variability in measurement positions, and the lack of standardized methodological designs make it difficult to translate these findings into clinical practice.

### 4.6. Practical Implications for Standardization

Current evidence indicates that musculoskeletal SWE is not only a feasible imaging method but also a tissue-specific quantitative measurement approach that requires careful standardization. Considering the anatomical characteristics of the examined structure, fiber orientation, position, loading condition, probe pressure, ROI selection, tissue depth, and technical features of the device, it is clear that SWE results cannot be interpreted independently of the measurement context. Therefore, the development of tissue-specific measurement protocols, common reporting standards, and inter-vendor harmonization are essential for the reliable integration of musculoskeletal SWE into clinical practice. Quantitative SWE data obtained with standardized measurement protocols have the potential to provide reliable and objective biomechanical information that may support clinical decision-making processes. 

A further limitation for clinical translation is the lack of validated cutoff values and clinically meaningful thresholds for most MSK-SWE applications. Reported thresholds are often specific to the tissue examined, disease stage, acquisition protocol, patient position, ROI definition, and SWE platform used. Therefore, proposed cutoff values should be interpreted cautiously and should not be generalized across populations, devices, or clinical settings without external validation. Future studies should determine whether SWE thresholds are associated with diagnostic accuracy, clinically relevant and patient-centered outcomes, functional measures, electrophysiology, MRI or histologic findings, treatment response, longitudinal responsiveness to rehabilitation, and return to activity.

### 4.7. Practical Advantages and Limitations of SWE Across Musculoskeletal Tissues

Although SWE is frequently described as a quantitative extension of musculoskeletal US, its clinical value differs substantially across tissue types. Conventional grayscale US primarily demonstrates morphology, including thickness, echotexture, fiber disruption, fascial thickening, nerve enlargement, or mass architecture, whereas Doppler US mainly provides information on vascularity and hyperemia. SWE adds a different dimension by estimating tissue stiffness and biomechanical behavior. This may be particularly useful when structural changes are subtle, when symptoms and morphological imaging findings are discordant, or when longitudinal monitoring of treatment or rehabilitation response is required. Compared with MRI, SWE has advantages related to real-time assessment, bedside availability, lower cost, repeatability, and the possibility of evaluating tissues under different positions or loading conditions. However, SWE should not be considered superior to MRI in general; rather, it may be advantageous in selected functional and biomechanical contexts, while MRI remains essential for comprehensive anatomical evaluation, deep structures, intra-articular pathology, bone marrow abnormalities, and preoperative assessment. The main tissue-specific advantages and disadvantages of SWE compared with conventional US, Doppler US, and MRI are summarized in Table 3.

### 4.8. Practical Clinical Indications for Requesting MSK-SWE

From a practical clinical perspective, MSK-SWE may be considered when quantitative biomechanical information is expected to contribute to diagnosis, severity assessment, treatment monitoring, or rehabilitation follow-up. The modality is most useful when conventional grayscale ultrasound or MRI demonstrates limited or nonspecific structural abnormalities; when symptoms and morphological imaging findings are discordant; or when repeated, dynamic, and bedside assessment of tissue stiffness is clinically relevant. The main clinical scenarios in which MSK-SWE may be considered are summarized in Table 4.

**Figure 2:**
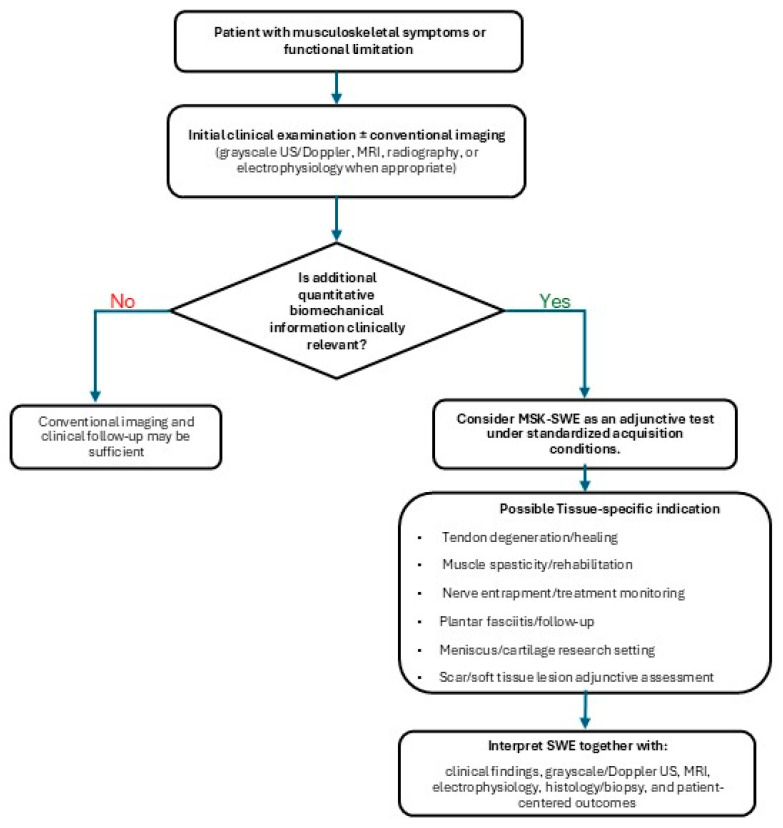
Practical decision flowchart for requesting MSK-SWE in clinical practice.

However, SWE should not be requested as a routine replacement for conventional imaging, electrophysiology, MRI, or histopathology. Instead, it should be used as an adjunctive technique in selected clinical scenarios and interpreted together with clinical findings, conventional imaging, and validated functional outcomes. Based on these clinical scenarios, a simplified decision pathway for non-radiologist clinicians is presented in Figure 2.

### 4.9. Tissue-Specific Pitfalls and Interpretation Cautions in MSK-SWE

The clinical impact of technical and biomechanical variables in MSK-SWE differs substantially according to the tissue being examined. A factor that is critical in one tissue may be less relevant in another. For example, anisotropy and fiber orientation are dominant sources of variability in tendons and ligaments, whereas muscle activation state, passive stretch, and joint position are more important in skeletal muscle assessment. In peripheral nerve imaging, stiffness values may be influenced by limb position, nerve tension, probe pressure, and discordance between cross-sectional area (CSA) and mechanical properties. In smaller or deeper structures such as the meniscus and articular cartilage, measurement depth, partial volume effects, and the adjacent bone interface may further reduce reliability. Similarly, in plantar fascia and soft tissue lesions, local tension, chronic loading, lesion heterogeneity, fibrosis, necrosis, or inflammation may complicate interpretation.

Therefore, SWE values should not be interpreted as isolated numerical measurements. They should be evaluated in relation to the anatomical structure, acquisition protocol, tissue depth, loading condition, device characteristics, and clinical context. For practical use, the main tissue-specific pitfalls and interpretation cautions are summarized in Table 5.

After these technical and practical considerations, the following sections summarize the main tissue-specific applications of SWE in musculoskeletal imaging. Selected original studies illustrating these applications are summarized in Table 6.

## 5. Clinical Applications of SWE in Musculoskeletal Imaging

### 5.1. SWE in Tendons and Ligaments

SWE is gaining increasing importance in musculoskeletal imaging because it can quantitatively assess the biomechanical properties of tendons and ligaments [7,66]. Although conventional US can demonstrate morphological changes such as tendon thickening, loss of fibrillar architecture, heterogeneity, and vascularity, it cannot directly characterize tissue stiffness. Therefore, SWE has emerged as a complementary method, particularly for detecting early degenerative changes, differentiating symptomatic from asymptomatic tendons, demonstrating load-related adaptive changes, and monitoring treatment response [41,67,68]. The literature has developed most extensively around the Achilles tendon, patellar tendon, rotator cuff tendons, and epicondylar tendons; among ligaments, the medial collateral ligament (MCL) and ulnar collateral ligament (UCL) have been the most frequently studied structures [69,70,71].

Tendon SWE findings are not unidirectional; pathological tendons have shown lower stiffness in some studies and higher stiffness in others [4,66]. This variability is largely related to the type of tendon examined, disease stage, anatomical segment, loading condition, and measurement protocol used [7]. Systematic reviews and meta-analyses suggest that shear wave velocity in tendinopathy may be lower than in healthy tendons; however, this finding should be interpreted with caution because of substantial methodological heterogeneity [7,66]. Conversely, higher stiffness values have been reported in the Achilles tendon of asymptomatic athletes and populations exposed to high mechanical loading. The observation that the Achilles tendon is stiffer in semiprofessional athletes and professional ballet dancers than in asymptomatic controls indicates that SWE measurements may reflect not only disease but also training-related physiological adaptation [39,72]. Although elastography findings may change in tendon injuries, no significant difference in Achilles tendon SWE values has been reported in primary hyperparathyroidism [6,73]. SWE has also been shown to have diagnostic value in patellar tendinopathy, with measurements from the proximal patellar tendon and those obtained at 30° knee flexion reported to provide higher diagnostic accuracy [40]. In addition, in vivo SWE studies comparing autograft tendons used in ACL reconstruction have demonstrated significant stiffness differences among the semitendinosus, quadriceps, and patellar tendons and have shown that variables such as age, sex, physical activity, and dominant side may affect tendon stiffness [40,67,70]. Taken together, these findings indicate that SWE in tendon assessment may reflect not only pathological changes but also normal variations related to tendon type, mechanical loading, and individual biomechanical characteristics. Therefore, the concepts of a “softer” or “stiffer” tendon should not be interpreted independently of the clinical and functional context.

In rotator cuff and epicondylar disorders, studies generally support a potential role for SWE in prognosis and treatment monitoring. Supraspinatus stiffness has been associated with symptomatic tears, repair outcomes, irreparability, and subclinical changes in diabetes, while longitudinal changes in tendon shear wave velocity have paralleled clinical improvement after treatment [41,42,43,68]. These findings suggest that SWE may capture clinically relevant mechanical changes beyond morphology. However, differences in patient populations, acquisition protocols, treatment methods, and follow-up intervals limit direct comparison and generalizability.

Studies on ligaments indicate that SWE is a potential method for providing quantitative and biomechanical information on ligamentous structures [44,71]. The definition of segmental stiffness values for the medial collateral ligament (MCL) in healthy volunteers represents an important starting point for future comparisons with traumatic or degenerative ligament disorders [69]. The finding of lower elasticity values and greater joint laxity in the dominant-side ulnar collateral ligament (UCL) of professional baseball players suggests that SWE may demonstrate biomechanical remodeling related to chronic loading [44]. However, cadaver-based mechanical correlation studies have found that SWE has limited ability to predict mechanical failure in superficial knee ligaments [74]. Therefore, although ligament SWE is a promising field, larger, prospective, and standardized studies are needed to support its clinical use.

Overall, the available evidence agrees that tendon and ligament SWE is sensitive to biomechanical state, loading history, and disease-related tissue changes. However, the direction of pathological stiffness change is not consistent across tendon types or disease stages, and evidence for ligaments remains less mature than that for tendons. The main remaining gaps are the absence of validated tissue-specific thresholds; limited cross-platform comparability; and insufficient longitudinal correlation with pain, function, return to sport, surgical healing, and treatment response.

### 5.2. SWE in Skeletal Muscles

SWE has become an important tool in musculoskeletal imaging because it can quantitatively assess the passive and active mechanical properties of skeletal muscle. Although conventional US can demonstrate morphological findings such as muscle thickness, echogenicity, atrophy, and fatty infiltration, it cannot directly evaluate the in vivo viscoelastic properties of muscle. Because SWE can quantitatively assess the stiffness of muscle tissue, it contributes to the objective evaluation of biomechanical states such as muscle tone, passive tension, contraction level, and spasticity [45,75]. It may also be used as a complementary imaging tool to demonstrate pain-related muscle changes and to monitor response to rehabilitation or injection therapies [8,76,77].

Measurement conditions have a marked effect on the results of muscle SWE. Muscle stiffness may change at rest, during passive stretching, and during active contraction; therefore, joint position, whether the muscle is relaxed or contracted, measurement depth, probe orientation, and patient position should be standardized. The variation in biceps brachii stiffness according to elbow position in healthy individuals shows that muscle SWE measurements are directly influenced by physiological position and passive tension [45]. Therefore, in muscle SWE, the biomechanical condition under which the measurement is obtained should be considered rather than relying on a single absolute value.

With regard to muscle injury, loading, and exercise-related changes, SWE has potential for monitoring the mechanical response of muscle tissue and stiffness changes during the healing process [78,79,80]. Studies showing that thigh muscle stiffness changes during rehabilitation after ACL injury and reconstruction suggest that SWE may be used to evaluate post-traumatic neuromuscular adaptations [81]. Similarly, the association of supraspinatus and infraspinatus muscle stiffness with reparability and surgical planning in rotator cuff tears indicates that SWE may provide prognostic information in evaluating the functional status of the musculotendinous unit [42,43]. However, because muscle SWE measurements may be affected by factors such as muscle activation state, joint position, loading history, and timing of measurement, the obtained values should be interpreted within standardized protocols and clinical context.

One of the strongest clinical applications of SWE in skeletal muscles is the assessment of spasticity. Spasticity developing after stroke, cerebral palsy, or traumatic brain injury may be associated with fibrosis, shortening, increased passive stiffness, and functional limitation in muscle tissue. Studies of the gastrocnemius medialis muscle in patients with chronic stroke have shown that SWE is feasible for evaluating the elastic properties of spastic muscles, although reliability is affected by measurement position and passive stretching condition [82]. In children with cerebral palsy, SWE has been reported to be useful for monitoring muscle stiffness and treatment response after botulinum toxin injection; in patients with spastic muscle overactivity after traumatic brain injury, increased shear wave velocity has been shown to be associated with the clinical severity of spasticity [8,46,76]. These data suggest that SWE may allow spasticity to be assessed objectively rather than only through subjective evaluation based on clinical scales.

SWE is also being investigated for assessing the relationship between muscle stiffness and symptom severity in chronic pain syndromes. In women with chronic nonspecific neck pain, despite perceived stiffness, objective stiffness of the neck extensor muscles was not different from that of controls, indicating that the subjective sensation of stiffness does not always correspond to measurable muscle stiffness [83]. In contrast, in chronic low back pain, quadratus lumborum stiffness has been associated with pain, central sensitization, and quality of life, and a reduction in resting stiffness of the lumbar erector spinae has been demonstrated after dry needling [47,84]. The decrease in masseter muscle stiffness after conservative treatment in temporomandibular disorders also supports the potential of SWE as an objective method for monitoring treatment response in myofascial and masticatory muscle disorders [85]. These findings indicate that the relationship between pain and muscle stiffness is not unidirectional and may vary according to muscle group, disease phenotype, and treatment type.

SWE is also used in the evaluation of postural and axial muscles. In idiopathic scoliosis, the elasticity of the lateral abdominal muscles has been reported to vary according to curve type and convex–concave side relationship, although findings have not always been consistent [86,87]. In a study investigating the relationship between vertebral fracture and trunk muscle stiffness in postmenopausal women, multifidus and erector spinae stiffness was found to be lower in some subgroups after controlling for bone mineral density [48]. These findings suggest that SWE may provide complementary information in the assessment of axial stability, postural control, and aging-related muscle quality. However, SWE-based cutoff values, normative data, and studies relating SWE findings to clinical outcomes remain insufficient for its integration into sarcopenia diagnostic algorithms.

Overall, SWE has considerable potential for the quantitative assessment of passive stiffness, activation level, spasticity, myofascial pain, exercise- or injury-related adaptations, and rehabilitation response in skeletal muscle. Findings are relatively consistent in demonstrating sensitivity to muscle activation, passive tension, and spasticity, whereas results in chronic pain, rehabilitation, postural disorders, and aging-related muscle changes remain more heterogeneous. The dynamic, viscoelastic, and contraction-sensitive nature of muscle tissue makes SWE measurements highly dependent on biomechanical and technical conditions. Therefore, standardized and reproducible measurement conditions are required, and the relationship of SWE values with clinical severity, functional status, patient-centered outcomes, and treatment response should be validated longitudinally.

### 5.3. SWE in Peripheral Nerves

SWE is increasingly used as a complementary method to conventional US in the evaluation of peripheral nerves. Although grayscale and Doppler US can demonstrate morphological and vascular features such as nerve thickness, increased CSA, disruption of fascicular architecture, changes in echogenicity, anatomical relationships with surrounding tissues, and vascularity, they cannot directly measure the mechanical properties of nerve tissue related to stiffness, tension, and compression [88,89]. SWE, by contrast, can quantitatively assess nerve elasticity and may therefore provide additional information in conditions such as entrapment neuropathies, diabetic peripheral neuropathy, and dynamic nerve tension [88,89,90,91,92]. The main aim of quantitative US approaches in peripheral nerve imaging is to reduce operator-dependent morphological interpretation and contribute to a more objective assessment of nerve disorders.

One of the most frequently investigated clinical applications of SWE in the peripheral nervous system is carpal tunnel syndrome (CTS) and median nerve assessment. In CTS, compression-related edema, increased intraneural pressure, fibrotic changes, and disruption of fascicular architecture in the median nerve may lead to measurable changes in nerve stiffness. Accordingly, studies combining high-frequency grayscale US and elastography have shown that the CSA and shear modulus of the median nerve increase gradually with CTS severity [49]. Similarly, evaluations performed at different wrist positions have reported that SWE may provide greater discriminatory value than CSA measurement in CTS grading and that median nerve stiffness measurements obtained at 45° wrist flexion may better reflect disease severity [50]. These findings suggest that SWE may contribute to diagnostic assessment and noninvasive severity grading in CTS.

CSA and SWE should therefore be interpreted as complementary rather than interchangeable parameters in CTS. CSA primarily reflects morphologic nerve enlargement, whereas SWE may provide additional information about mechanical changes related to compression, intraneural pressure, edema, fibrosis, or altered nerve tension. Reliance on CSA alone may lead to false-positive interpretation or overdiagnosis in patients with anatomical variation, borderline nerve enlargement, obesity-related soft tissue changes, or discordance between imaging findings and symptoms. In such situations, SWE may add discriminatory value by providing biomechanical information that is not captured by morphologic measurement alone. Combined CSA–SWE assessment may therefore be more clinically meaningful than reliance on a single parameter, particularly when interpreted together with symptoms, physical examination, and electrophysiological findings. Future CTS studies should report diagnostic performance metrics, including sensitivity, specificity, positive predictive value, negative predictive value, area under the curve, and severity-stratified thresholds for CSA alone, SWE alone, and combined CSA–SWE models under standardized acquisition conditions [49,50,51,93].

Position and mechanical loading are important determinants in median nerve SWE measurements. In an ARFI elastography study conducted in healthy individuals, shear wave velocity values of the median nerve, flexor tendons, and transverse carpal ligament changed significantly according to wrist and finger positions [93]. This finding shows that structures within the carpal tunnel exhibit different mechanical responses in neutral and stress positions, and therefore, nerve elasticity measurements should not be interpreted as static or context-independent values. For this reason, standardization of variables such as wrist angle, finger position, probe pressure, and ROI placement is essential for measurement reliability in CTS studies.

Another important application of SWE is monitoring treatment response. In patients with CTS, significant decreases in median nerve CSA, shear wave velocity, and Young’s modulus have been observed after ultrasound-guided local steroid injection, accompanied by marked improvement in clinical symptom scores [51]. This finding suggests that SWE may objectively demonstrate biomechanical improvement in nerve tissue after treatment and may provide a quantitative parameter that can be used not only at the diagnostic stage but also during follow-up. However, prospective multicenter studies with longer follow-up periods, comparisons of different treatment modalities, and correlation with electrophysiological outcomes are needed in this field.

Studies on the ulnar nerve also show that SWE is applicable to different types of entrapment neuropathy. In ulnar tunnel syndrome at the level of Guyon’s canal, ulnar nerve stiffness has been found to be significantly higher than in healthy controls, supporting the measurable effect of focal compression on nerve elasticity [53]. Dynamic evaluations at the elbow have shown that valgus stress increases ulnar nerve stiffness, with this effect being more pronounced at proximal entrapment sites [94]. These findings indicate that peripheral nerve SWE may contribute not only to static morphological assessment but also to the evaluation of nerve biomechanics related to tension, valgus loading, and functional positions.

An intervention-oriented application of peripheral nerve SWE has also been reported in post-surgical radial nerve entrapment. Su et al. described two cases of radial nerve injury after humeral shaft fracture fixation in which SWE was used to identify hardened scar tissue surrounding the nerve and to define targets for ultrasound-guided perineural hydrodissection [95]. This example suggests that SWE may contribute not only to diagnosis or follow-up but also to procedural planning by helping localize mechanically abnormal perineural tissue in selected post-surgical neuropathies. However, this evidence is currently limited to case reports, and larger studies are needed before this approach can be generalized.

Diabetic peripheral neuropathy (DPN) is another area in which peripheral nerve SWE is being increasingly investigated clinically. In patients with type 2 diabetes, tibial nerve stiffness has been shown to increase significantly in the presence of DPN, and the combined use of SWE with conventional US has been reported to improve diagnostic performance [52]. Another prospective study showed that the tibial nerve could be evaluated at both the ankle and popliteal fossa levels, although SWE measurements at the ankle level may provide a stronger contribution to the diagnosis of DPN [96]. These data suggest that SWE is promising, particularly for detecting subclinical or early-stage DPN and for quantitatively monitoring peripheral nerve involvement.

In conclusion, current evidence consistently supports the complementary value of peripheral nerve SWE in CTS and selected peripheral neuropathies. Agreement is stronger for the detection of increased or position-dependent nerve stiffness than for universal diagnostic thresholds or severity grading. Peripheral nerve SWE should therefore be interpreted together with morphologic ultrasound, CSA, symptoms, and electrophysiological findings rather than as a replacement for these assessments. The main remaining gaps are the lack of standardized acquisition protocols; externally validated combined CSA–SWE models; and longitudinal validation against symptoms, electrophysiological findings, functional outcomes, and treatment response.

### 5.4. SWE in Menisci, Cartilage, and Other Emerging Applications

SWE has also become an increasingly investigated area in musculoskeletal structures beyond the conventional tendon–muscle–nerve axis, including the meniscus, plantar fascia, annulus fibrosus, and soft tissue masses [13,54,55,60,62,97]. Although conventional US and MRI are the main imaging methods for evaluating findings such as structural disruption, increased thickness, signal change, and mass morphology, they do not provide direct quantitative information on the elastic properties of tissues. In this respect, SWE is considered a complementary method that can add biomechanical information to morphological imaging in meniscal degeneration, plantar fascia disorders, axial fibrocartilaginous structures, and soft tissue lesions.

#### 5.4.1. Menisci

The meniscus is a fibrocartilaginous structure with a complex collagen architecture that plays a key role in load transmission, shock absorption, and joint stability. Across meniscal studies, higher SWE values have generally been associated with histologic or MRI-defined degeneration and with clinical factors such as age, BMI, obesity, diabetes, and osteoarthritis [54,55,56,97,98,99]. This consistency supports the sensitivity of SWE to biomechanical changes associated with meniscal degeneration. However, differences between medial and lateral menisci, patient populations, disease stages, and reference standards limit direct comparison. The small size, deep location, curved anatomy, regional collagen orientation, and proximity to bone also make measurements sensitive to ROI depth, probe selection, and measurement plane [100]. The main remaining gaps are the absence of standardized meniscal segments; externally validated thresholds; and longitudinal evidence showing whether SWE predicts symptoms, progression, or treatment response.

#### 5.4.2. Articular Cartilage and Annulus Fibrosus

For articular cartilage, SWE remains an early-stage application. The thin structure of cartilage, its proximity to the subchondral bone interface, and its load-sensitive biomechanics may limit the reliability of direct SWE measurements. Although current studies suggest that cartilage elasticity measurements may have potential to reflect early biomechanical changes related to osteoarthritis, clinical evidence in this field remains limited, and larger standardized studies are needed [57,58].

In terms of fibrocartilaginous and axial structures, annulus fibrosus SWE represents another developing application. The finding that lumbar annulus fibrosus shear wave speed values are higher in adolescents with idiopathic scoliosis than in healthy individuals suggests that the mechanical properties of intervertebral disc tissue may be evaluated noninvasively with SWE [59]. Similarly, the observation that lumbar annulus fibrosus stiffness values are higher after fusionless bipolar fixation in neuromuscular scoliosis than in healthy controls indicates that SWE may serve as a complementary tool for evaluating spinal biomechanical changes [101]. However, studies in this area remain limited; larger, prospective, and standardized studies are needed to determine the contribution of annulus fibrosus SWE to clinical decision-making.

#### 5.4.3. Plantar Fascia

The plantar fascia represents a distinct application area for SWE because it is a superficial, load-bearing fibrous structure that is readily evaluated by US in patients with heel pain and suspected plantar fasciitis. Plantar fascia studies generally agree that SWE adds biomechanical information to conventional measurements of thickness and echotexture and may support the diagnosis and monitoring of plantar fasciitis [61,102,103]. Diagnostic studies have reported promising performance, while treatment studies have demonstrated stiffness changes associated with clinical improvement. However, findings in asymptomatic runners indicate that chronic loading alone does not necessarily produce the same SWE changes as symptomatic disease [104]. This difference emphasizes that plantar fascia stiffness should be interpreted together with symptoms, loading history, foot position, and conventional US findings. The principal remaining gaps are the lack of externally validated thresholds and limited longitudinal correlation with pain, function, recurrence, and sustained treatment response [105].

#### 5.4.4. Soft Tissue Masses, Scar, Bursa, and Synovium

Evidence on SWE in other musculoskeletal applications, such as soft tissue masses, scar/fibrosis, bursa, and synovium, is more limited and heterogeneous. In soft tissue tumors, some studies have reported that real-time SWE parameters may contribute to benign–malignant differentiation, particularly measurements reflecting intralesional elasticity heterogeneity, which may be associated with malignancy [62]. However, larger prospective series have shown that SWE alone does not provide a sufficient diagnostic criterion to reliably differentiate benign from malignant soft tissue masses [31,37,63]. Therefore, lesion size, depth, vascularity, MRI features, and histopathological confirmation remain decisive in the diagnostic process. In soft tissue masses, SWE should be considered a complementary parameter that can provide additional biomechanical information to conventional US, rather than an independent method that replaces biopsy or MRI. For structures such as scar/fibrosis, bursa, and synovium, current data remain at an earlier stage [64,65,106]. Although SWE has theoretical potential for the quantitative assessment of fibrotic stiffening, inflammatory thickening, or post-treatment tissue response in these tissues, sufficient standardization and validation to support routine clinical use are not yet available.

In conclusion, the meniscus and plantar fascia provide a stronger basis in terms of their relationship with clinical and imaging findings, whereas articular cartilage, annulus fibrosus, soft tissue masses, scar/fibrosis, bursa, and synovium remain among more developing applications. Current data indicate that, in these areas, SWE should be considered not as a standalone method for clinical decision-making but as a complementary tool that may provide additional information to morphological assessment. Therefore, for reliable clinical interpretation in these applications, it is important to establish tissue-specific measurement reliability and to comparatively evaluate SWE findings against independent references such as MRI, histopathology, clinical scores, or treatment response.

## 6. Future Directions

To define the clinical role of MSK-SWE more clearly, future studies should focus on the key knowledge gaps that currently limit its translation into routine practice. First, tissue-specific measurement protocols are needed because tendons, muscles, nerves, menisci, plantar fascia, cartilage, and other fibrocollagenous structures differ in anisotropy, viscoelasticity, depth, and loading sensitivity. Therefore, patient position, joint angle, probe orientation, ROI placement, number of repeated measurements, and reported parameters should be standardized separately for each tissue.

Second, normative reference ranges and validated clinical thresholds remain insufficient for most musculoskeletal tissues. Because age, sex, body mass index, physical activity level, dominant side, and anatomical variations may affect SWE measurements, reference values should be established in well-defined healthy populations and validated in independent clinical cohorts. In addition, proposed cutoff values should be tested across different devices and populations before they are used for clinical decision-making.

Third, inter-vendor harmonization remains a major methodological gap. Acoustic radiation force settings, shear wave tracking algorithms, elastogram reconstruction methods, presets, and output parameters differ among manufacturers. Phantom studies and comparative clinical studies performed on the same tissues using different SWE platforms are needed to clarify when values reported in m/s or kPa can be compared and when device-specific interpretation is required.

From a clinical perspective, future studies should evaluate SWE not only as a marker of tissue stiffness but also as a tool linked to meaningful clinical outcomes. Longitudinal studies in tendinopathies, peripheral nerve entrapments, muscle spasticity, plantar fascia disorders, and meniscal or cartilage degeneration should correlate SWE changes with pain, function, electrophysiology, MRI, histology, surgical findings, treatment response, and return-to-activity outcomes. Peripheral nerve studies may incorporate symptom and functional scales such as the Boston Carpal Tunnel Questionnaire, whereas tendon, plantar fascia, and knee-related studies may include validated instruments such as DASH/QuickDASH, VISA-A/VISA-P, FAAM, WOMAC, KOOS, or other disease-specific outcome measures. Such outcome-based validation is necessary to determine whether SWE can meaningfully influence treatment selection, rehabilitation monitoring, or clinical decision-making.

Finally, artificial intelligence-assisted image analysis, automated ROI selection, quality control algorithms, and multiparametric US approaches may improve the reproducibility and clinical applicability of MSK-SWE. The combined use of SWE with Doppler or microvascular imaging, grayscale morphometry, and clinical scores may allow tissue disorders to be assessed through mechanical, structural, vascular, and functional information together. However, these approaches should be validated against clinically meaningful endpoints before routine clinical use.

## 7. Conclusions

SWE is a method that complements the morphological findings obtained by conventional US in musculoskeletal imaging with quantitative biomechanical data. Studies in different musculoskeletal structures, including tendons, ligaments, skeletal muscles, peripheral nerves, menisci, and the plantar fascia, indicate that SWE has potential for detecting early biomechanical changes, assessing disease severity, and monitoring treatment response. However, the anisotropic and viscoelastic nature of musculoskeletal tissues makes SWE measurements susceptible to many technical and biological variables. Therefore, SWE findings should not be interpreted as independent standalone diagnostic criteria, but rather in conjunction with clinical findings, conventional US, MRI, electrophysiology, or histopathological data.

In conclusion, SWE is a promising biomechanical imaging method that is progressing from the research setting toward clinical application in musculoskeletal imaging. However, its true clinical impact will become clearer with tissue-specific standardization, improved inter-vendor harmonization, and reliable correlation of quantitative measurements with functional, rehabilitation-related, and patient-centered clinical outcomes.

## Figures and Tables

**Table 1:** Comparison of elastography techniques used in musculoskeletal imaging.

Technique	Excitation Method	Output Parameter	Operator Dependency	Main Advantages	Main Limitations in MSK Imaging
Strain/compression elastography	Manual compression or physiological motion	Relative strain, color map, strain ratio	High	Simple, widely available, useful for qualitative stiffness assessment	Semiquantitative; strongly affected by compression technique and operator experience
ARFI imaging	Focused acoustic radiation force impulse	Tissue displacement or localized stiffness-related response	Moderate	Less dependent on manual compression; can assess deeper tissues than manual strain techniques	Small sampling region; device-specific algorithms; limited comparability
Point SWE	Acoustic radiation force-generated shear waves	Shear wave velocity, usually m/s	Moderate	Quantitative measurement from a defined region; useful for focal assessment	Limited spatial mapping; sensitive to ROI placement, depth, and tissue anisotropy
2D-SWE	Acoustic radiation force-generated shear waves with ultrafast tracking	Color-coded stiffness map, m/s and/or kPa	Moderate	Provides quantitative and spatial stiffness information over a larger field of view	Sensitive to artifacts, anisotropy, motion, probe pressure, and inter-vendor variability

Abbreviations: ARFI, acoustic radiation force impulse; MSK, musculoskeletal; ROI, region of interest; SWE, shear wave elastography.

**Table 2:** Technical factors affecting SWE measurements in musculoskeletal tissues.

Factor	Effect on Measurement	Practical Recommendation
Anisotropy	Fiber orientation alters stiffness values.	Scan in a standardized plane, preferably along the long axis of fibers.
Probe pressure	Excessive compression may falsely increase stiffness.	Use minimal, consistent pressure and adequate gel.
Joint position	Joint angle changes passive tissue tension.	Standardize and report the exact joint position.
Muscle activation	Contraction increases stiffness values.	Ensure relaxation unless contraction is being specifically assessed.
ROI placement	ROI size/location affects values, especially in small or heterogeneous tissues.	Place ROI within homogeneous target tissue and avoid borders or adjacent structures.
Depth	Greater depth may reduce signal quality and reliability.	Avoid excessive depth when possible and report measurement depth.
Vendor variability	Different systems may produce non-equivalent values.	Use the same device, probe, preset, and software for follow-up.
Operator experience	Inconsistent technique reduces reproducibility.	Use trained operators and standardized acquisition protocols.

Abbreviations: ROI, region of interest; SWE, shear wave elastography.

**Table 3:** Advantages and disadvantages of SWE according to musculoskeletal tissue.

Tissue/Structure	Potential Advantages of SWE	Advantages Compared with Grayscale US/Doppler	Potential Advantages Compared with MRI	Main Disadvantages/Limitations
Tendons and ligaments	Quantifies stiffness, loading-related adaptation, degeneration, and treatment response.	Grayscale US shows thickening, fibrillar disruption, and tears; Doppler shows hyperemia. SWE adds biomechanical stiffness information.	Allows real-time and dynamic assessment under different joint positions or loading states.	Strongly affected by anisotropy, tendon tension, probe orientation, joint position, and loading history.
Skeletal muscles	Assesses passive stiffness, contraction-related stiffness, spasticity, and rehabilitation response.	Grayscale US shows thickness, echogenicity, atrophy, and fatty change; Doppler has limited value for mechanical assessment. SWE directly reflects muscle mechanical state.	May be more practical than MRI for repeated bedside functional assessment, especially during passive stretch or contraction.	Highly dependent on muscle activation, passive stretch, joint angle, depth, and patient cooperation.
Peripheral nerves	Evaluates nerve stiffness related to compression, fibrosis, tension, or treatment response.	Grayscale US mainly measures CSA and fascicular morphology. SWE may add information when CSA is borderline or nonspecific.	Allows dynamic assessment of nerve tension in different limb positions and may support treatment monitoring.	Influenced by limb position, nerve tension, ROI size, probe pressure, and adjacent tendons or vessels.
Menisci	May detect biomechanical changes associated with degeneration before or alongside structural imaging findings.	Conventional US has limited ability to characterize internal meniscal biomechanics. SWE may provide quantitative stiffness information.	Potentially useful as a quick adjunct for superficial meniscal segments, especially in research settings.	Small size, depth, curved anatomy, bone interface, and regional collagen orientation limit reliability.
Articular cartilage	May reflect early biomechanical cartilage alterations.	Grayscale US mainly evaluates surface and thickness in accessible regions; SWE may add stiffness-related information.	Could provide functional information complementary to MRI compositional imaging.	Thin structure, subchondral bone interface, loading sensitivity, and limited validation restrict clinical use.
Plantar fascia	Quantifies stiffness changes in plantar fasciitis and after treatment.	Grayscale US shows fascia thickening and echogenicity; SWE adds stiffness and healing-related biomechanical information.	More accessible, repeatable, and dynamic for follow-up than MRI in typical heel pain evaluation.	Affected by probe pressure, foot position, fascia tension, chronic loading, and symptom–imaging discordance.
Soft tissue masses, scar, synovium, and bursa	May characterize tissue stiffness, fibrosis, or heterogeneity as an adjunctive parameter.	Adds biomechanical information to morphology and vascularity.	May help guide further evaluation but cannot replace MRI for lesion extent or staging.	Considerable overlap between benign and malignant lesions; should not replace MRI, biopsy, or histopathology.

Abbreviations: CSA, cross-sectional area; MRI, magnetic resonance imaging; ROI: region of interest; SWE, shear wave elastography; US, ultrasonography.

**Table 4:** Clinical scenarios, indications, and practical value of MSK-SWE.

Clinical Scenario	When SWE May Be Indicated	Practical Clinical Value
Tendinopathy or tendon pain	Persistent tendon pain, suspected early degeneration, symptom–US discordance, or follow-up after treatment.	Adds quantitative stiffness information that may support assessment of degeneration, load-related adaptation, or healing.
Post-treatment tendon follow-up	After PRP, corticosteroid injection, rehabilitation, or surgery when biomechanical recovery is clinically relevant.	May help monitor stiffness changes alongside pain and functional scores.
Muscle spasticity or abnormal tone	Stroke, cerebral palsy, traumatic brain injury, or other conditions with suspected increased muscle stiffness.	May provide objective quantification of passive muscle stiffness and may support treatment planning or botulinum toxin/rehabilitation follow-up.
Muscle rehabilitation or sports injury	Monitoring recovery after muscle injury, ACL reconstruction, low back pain rehabilitation, or dry needling.	Enables repeated assessment of muscle mechanical response under standardized conditions.
Peripheral nerve entrapment	CTS, ulnar neuropathy, post-surgical scarring, or suspected nerve compression when CSA or symptoms are inconclusive.	Adds stiffness-related information to nerve morphology and may support severity grading or treatment monitoring.
Diabetic peripheral neuropathy	Patients with diabetes and suspected early or subclinical peripheral nerve involvement.	May contribute to quantitative assessment of nerve stiffness when combined with conventional US and clinical/electrophysiological findings.
Plantar fasciitis/heel pain	Heel pain with suspected plantar fasciitis or follow-up after injection/rehabilitation.	Adds stiffness data to fascia thickness and echotexture and may help monitor treatment response.
Meniscal or cartilage degeneration	Research setting or selected cases where early biomechanical degeneration is being investigated.	May provide adjunctive biomechanical information, but clinical cutoffs remain insufficiently validated.
Soft tissue masses or scar/fibrosis	Selected lesions where stiffness or heterogeneity may provide adjunctive information.	May supplement conventional US, but should not replace MRI, biopsy, or histopathology.

Abbreviations: ACL, anterior cruciate ligament; CSA, cross-sectional area; CTS, carpal tunnel syndrome; MSK-SWE, musculoskeletal shear wave elastography; MRI, magnetic resonance imaging; PRP, platelet-rich plasma; SWE, shear wave elastography; US, ultrasonography.

**Table 5:** Tissue-related technical factors affecting MSK-SWE interpretation and practical considerations.

Tissue/Structure	Main Pitfalls	Potential Effect on SWE Interpretation	Practical Caution
Tendons and ligaments	Anisotropy, non-parallel probe orientation, variable joint position, passive tension, loading history, tendon segment differences.	May cause falsely high or low stiffness values and poor comparability between studies.	Use a standardized longitudinal plane, report joint position and tendon segment, and avoid interpreting stiffness as uniformly pathological.
Skeletal muscles	Active or involuntary contraction, passive stretch, joint angle, measurement depth, pennation angle, patient cooperation.	Muscle stiffness may reflect contraction or stretch rather than disease-related tissue alteration.	Specify whether the muscle is measured at rest, during passive stretch, or contraction; standardize limb position.
Peripheral nerves	Limb and joint position, nerve tension, probe pressure, small ROI, adjacent tendons or vessels, CSA–stiffness discordance.	Stiffness may vary dynamically and may not directly correspond to nerve enlargement or symptom severity.	Interpret SWE with CSA, clinical symptoms, electrophysiology, and standardized wrist/elbow/limb positioning.
Menisci	Small size, deep location, curved anatomy, bone interface, regional collagen orientation, ROI depth.	Measurements may be affected by partial volume effects and limited acoustic window.	Use standardized meniscal segment and ROI placement; interpret mainly as adjunctive or research-oriented information.
Articular cartilage	Thin structure, subchondral bone interface, load sensitivity, limited accessible surface, partial volume effects.	Bone-related artifacts and small ROI errors may reduce reliability.	Avoid overinterpreting isolated cartilage stiffness values; correlate with MRI, symptoms, and osteoarthritis grading.
Plantar fascia	Probe pressure, foot position, fascia tension, chronic loading, symptom–imaging mismatch.	Stiffness changes may reflect loading or tension rather than active plantar fasciitis alone.	Standardize ankle/foot position and interpret with fascia thickness, echotexture, and pain/function scores.
Soft tissue masses, scar, synovium, and bursa	Lesion heterogeneity, necrosis, fibrosis, inflammation, depth, vascularity, overlap between benign and malignant lesions.	SWE may not reliably distinguish benign from malignant lesions when used alone.	Use SWE only as an adjunct; do not replace MRI, biopsy, histopathology, or oncologic assessment.

Abbreviations: CSA, cross-sectional area; MRI, magnetic resonance imaging; SWE, shear wave elastography; ROI, region of interest.

**Table 6:** Selected original studies illustrating tissue-specific applications of SWE in musculoskeletal imaging.

Author, Year	Study Design/Type	Tissue/Pathology	Study Population	Key SWE Findings	Clinical Implication	Main Limitation
Dirrichs et al., 2019 [39]	Cross-sectional comparative study	Achilles tendon stiffness	Semiprofessional athletes and nonathletic healthy individuals	Healthy athletes showed higher Achilles tendon SWE values than nonathletic participants.	Provides reference data on activity-related Achilles tendon stiffness and highlights the influence of loading history.	Asymptomatic population; does not directly evaluate tendinopathy or treatment response.
Devran et al., 2024 [40]	Diagnostic accuracy/cross-sectional study	Patellar tendinopathy	Female volleyball and basketball athletes	Diagnostic performance varied according to tendon segment and knee angle.	Measurement site and joint position should be standardized when assessing patellar tendon stiffness.	Sport- and sex-specific cohort.
Stewart et al., 2026 [41]	Randomized controlled trial	Lateral epicondylosis after PRP	Patients with lateral epicondylosis treated with PRP or comparator treatment	Tendon shear wave changes were associated with healing and clinical improvement.	SWE may provide an objective marker of treatment response in lateral epicondylosis.	Protocol-specific findings; limited generalizability to other treatments.
Jeong et al., 2022 [42]	Clinical cohort study with MRI correlation	Supraspinatus muscle/rotator cuff tear	Patients undergoing rotator cuff repair with MRI correlation	Preoperative supraspinatus muscle stiffness was associated with repair-related outcomes.	SWE may support preoperative assessment and surgical planning in rotator cuff disease.	Single-pathology focus; technique-dependent measurements.
Itoigawa et al., 2026 [43]	Preoperative clinical study	Large or massive rotator cuff tears	Patients evaluated before surgery	SWE showed value in predicting irreparability of large or massive rotator cuff tears.	May complement MRI in presurgical planning for rotator cuff repair.	Findings require validation across devices and surgical cohorts.
Yoshikawa et al., 2022 [44]	Cross-sectional athlete study	Ulnar collateral ligament of the elbow	Professional baseball players	UCL stiffness could be assessed with SWE in throwing athletes.	May help characterize ligament adaptation or injury risk in overhead athletes.	Athlete-specific cohort; lack of broad clinical validation.
Chen et al., 2017 [45]	Experimental cross-sectional study	Biceps brachii stiffness	Healthy volunteers	Passive stiffness differed according to elbow position.	Highlights the importance of joint position standardization in muscle SWE.	Small healthy cohort.
Matsumoto-Miyazaki et al., 2022 [46]	Observational comparative study	Spastic biceps brachii	Patients after severe traumatic brain injury	Shear wave speed was higher than in controls and correlated with spasticity severity.	SWE may quantify the mechanical component of spasticity.	Small observational cohort.
Koppenhaver et al., 2022 [47]	Randomized/sham-controlled interventional study	Lumbar multifidus and erector spinae	Patients with low back pain undergoing dry needling	Resting erector spinae stiffness decreased after dry needling compared with sham.	SWE may monitor short-term muscle response to rehabilitation interventions.	Short-term follow-up.
Bayraktar et al., 2026 [48]	Cross-sectional study	Trunk muscles and vertebral fracture	Postmenopausal women aged ≥60 years	Multifidus and erector spinae stiffness differed in some vertebral fracture subgroups after BMD adjustment.	May contribute to assessment of trunk muscle quality and spinal stability.	Cross-sectional design.
Prakash et al., 2024 [49]	Diagnostic case–control study	Median nerve; carpal tunnel syndrome	CTS patients and healthy controls	Median nerve CSA and shear modulus increased with CTS severity.	SWE may complement grayscale US for CTS grading.	Single-center design.
Zou et al., 2025 [50]	Diagnostic cross-sectional study	Median nerve; CTS at different wrist angles	CTS patients and healthy controls	SWE showed better severity discrimination at specific wrist positions.	Wrist position should be standardized when using SWE for CTS assessment.	Position-specific cutoffs may not generalize.
Gürün et al., 2025 [51]	Prospective interventional follow-up study	Median nerve after steroid injection	Patients with mild-to-moderate CTS	CSA, SWV, and Young’s modulus decreased after injection with clinical improvement.	SWE may objectively monitor treatment response after US-guided steroid injection.	Short-term follow-up; no untreated control group.
Peng et al., 2025 [52]	Diagnostic observational study	Tibial nerve; diabetic peripheral neuropathy	Type 2 diabetes patients	Tibial nerve stiffness was higher in DPN; combined US and SWE improved diagnostic performance.	May support early and more consistent assessment of DPN.	Reader experience and protocol dependence.
Paluch et al., 2018 [53]	Case–control diagnostic study	Ulnar nerve; ulnar tunnel syndrome	Patients with ulnar tunnel syndrome and controls	SWE showed potential for detecting abnormal ulnar nerve stiffness.	Extends peripheral nerve SWE beyond CTS.	Limited disease-specific evidence and need for larger validation cohorts.
Park et al., 2020 [54]	Histology-correlated clinical study	Meniscus degeneration	TKA patients with histologic reference	Meniscus stiffness increased with histologic degeneration.	SWE may provide biomechanical assessment of meniscal degeneration.	Small sample; advanced OA population.
Gürün et al., 2021 [55]	MRI-correlated clinical study	Meniscus degeneration	Patients with MRI correlation	Meniscus stiffness correlated with MRI degeneration grade and age.	May complement MRI in grading meniscal degeneration.	MRI rather than histology as reference.
Dag et al., 2024 [56]	Cross-sectional comparative study	Meniscus stiffness in obesity	Obese children/adolescents and controls	Meniscal stiffness was higher in obese children and adolescents.	May detect early biomechanical meniscal effects of pediatric obesity.	Preliminary cross-sectional design.
Kaplan et al., 2023 [57]	Imaging correlation study	Trochlear cartilage damage	Patients evaluated with SWE and T2-star mapping	SWE showed potential for detecting early-stage trochlear cartilage damage.	May complement MRI-based cartilage assessment in early cartilage degeneration.	Preliminary design; limited cartilage-specific validation.
Yokus et al., 2021 [58]	Preliminary clinical study	Distal femoral cartilage; knee osteoarthritis	Patients with knee OA	Femoral cartilage was assessed using grayscale US and SWE.	SWE may add biomechanical information to conventional cartilage evaluation.	Preliminary study; technical challenges in thin cartilage assessment.
Langlais et al., 2018 [59]	Comparative clinical study	Lumbar annulus fibrosus; adolescent scoliosis	Adolescents with scoliosis	SWE could detect annulus fibrosus alteration.	Suggests a potential role for SWE in assessing spinal soft tissue biomechanics.	Emerging application; limited standardization.
Vergari et al., 2020 [60]	Pre-/postoperative clinical study	Lumbar annulus fibrosus before and after scoliosis surgery	Adolescents undergoing surgical intervention	SWE demonstrated changes in annulus fibrosus stiffness after surgery.	May help evaluate biomechanical effects of spinal deformity correction.	Specialized cohort; limited generalizability.
Ramu et al., 2023 [61]	Case–control diagnostic study	Plantar fasciitis	Patients with plantar fasciitis and controls	SWE helped differentiate plantar fasciitis from healthy plantar fascia.	May complement grayscale US in heel pain evaluation.	Single-condition case–control design.
Li et al., 2020 [62]	Diagnostic observational study	Musculoskeletal soft tissue tumors	Patients with benign and malignant soft tissue tumors	Real-time SWE parameters differed between benign and malignant lesions.	May supplement conventional US in soft tissue mass assessment.	Overlap between tumor types.
Winn et al., 2020 [63]	Prospective musculoskeletal oncology cohort	Soft tissue tumors	Patients referred to an musculoskeletal oncology setting	SWE did not provide a reliable benign–malignant threshold.	SWE should not replace MRI or biopsy in soft tissue tumor characterization.	Lesion heterogeneity.
DeJong et al., 2020 [64]	Reliability/protocol development study	Scar stiffness	Patients with scar tissue assessment	A reliable SWE-based protocol for scar stiffness assessment was described.	Provides an objective method for quantifying scar stiffness.	Requires broader clinical validation and outcome correlation.
Chandel et al., 2022 [65]	Comparative diagnostic study	Synovium; rheumatoid versus tubercular arthritis	Patients with inflammatory arthritis	Synovial SWE differed between rheumatoid and tubercular arthritis.	May provide adjunctive information in inflammatory synovial disease.	Small, disease-specific cohort; emerging application.

Abbreviations: BMD, bone mineral density; CSA, cross-sectional area; CTS, carpal tunnel syndrome; DPN, diabetic peripheral neuropathy; MRI, magnetic resonance imaging; OA, osteoarthritis; PRP, platelet-rich plasma; SWE, shear wave elastography; SWV, shear wave velocity; TKA, total knee arthroplasty; UCL, ulnar collateral ligament.

## Data Availability

No new data were created or analyzed in this study. Data sharing is not applicable to this article.

## Supplementary Materials

The following supporting information can be downloaded at: https://www.mdpi.com/article/10.3390/jcm15124843/s1, Supplementary Table S1: Literature search strategy.

## Abbreviations

The following abbreviations are used in this manuscript:

SWE	Shear wave elastography
MSK-SWE	Musculoskeletal shear wave elastography
US	Ultrasonography
ROI	Region of interest
SWV	Shear wave velocity
kPa	Kilopascal
MRI	Magnetic resonance imaging
DPN	Diabetic peripheral neuropathy
CTS	Carpal tunnel syndrome
