# Supplementary material for: Shear Wave Elastography in Musculoskeletal Imaging: A Narrative Review
Source: J Clin Med. 2026 Jun 22;15(12):4843. doi: 10.3390/jcm15124843 (PMC13301753; doi:10.3390/jcm15124843)
Supplement: Supplementary file 1 [file jcm-15-04843-s001.zip › jcm-4346858-supplementary.pdf]

Supplementary Table S1. Literature search strategy

Item	Description
Review type	Narrative review
Main database	PubMed/MEDLINE
Additional source	Reference lists of selected original articles, review articles, systematic reviews, technical papers, and guideline/consensus documents
Search period	Articles published within the last 10 years up to May 2026
Main search concept 1	"shear wave elastography" OR "sonoelastography" OR "elasticity imaging techniques" OR "acoustic radiation force impulse" OR "point shear wave elastography" OR "two-dimensional shear wave elastography"
Main search concept 2	"musculoskeletal" OR "musculoskeletal imaging" OR "tendon" OR "ligament" OR "skeletal muscle" OR "peripheral nerve" OR "carpal tunnel syndrome" OR "meniscus" OR "cartilage" OR "plantar fascia" OR "soft tissue mass"
Main search concept 3	"technical considerations" OR "artifact" OR "standardization" OR "reproducibility" OR "reliability" OR "diagnostic accuracy" OR "treatment monitoring"
Example Boolean search string	("shear wave elastography" OR "sonoelastography" OR "elasticity imaging techniques" OR "acoustic radiation force impulse" OR "point shear wave elastography" OR "two-dimensional shear wave elastography") AND ("musculoskeletal" OR "musculoskeletal imaging" OR "tendon" OR "ligament" OR "skeletal muscle" OR "peripheral nerve" OR "carpal tunnel syndrome" OR "meniscus" OR "cartilage" OR "plantar fascia" OR "soft tissue mass") AND ("technical considerations" OR "artifact" OR "standardization" OR "reproducibility" OR "reliability" OR "diagnostic accuracy" OR "treatment monitoring")
Inclusion focus	Original research articles, prospective and retrospective clinical studies, diagnostic accuracy studies, interventional follow-up studies, reliability studies, technical papers, systematic reviews, narrative reviews, scoping reviews, and guideline/consensus documents relevant to musculoskeletal SWE
Selection approach	Articles were selected according to clinical relevance, methodological contribution, tissue-specific representativeness, and ability to illustrate key technical or clinical aspects of MSK-SWE
Synthesis method	Qualitative narrative synthesis; no quantitative meta-analysis was performed
